# Ti_3_AlC_2_ MAX Phase Modified Screen-Printed Electrode for the Fabrication of Hydrazine Sensor

**DOI:** 10.3390/mi15050633

**Published:** 2024-05-09

**Authors:** Khursheed Ahmad, Waseem Raza, Rais Ahmad Khan

**Affiliations:** 1 School of Materials Science and Engineering, Yeungnam University, Gyeongsan 38541, Republic of Korea; 2 Department of Materials Science and Engineering, WW4-LKO, University of Erlangen-Nuremberg, Martensstrasse 7, 91058 Erlangen, Germany; 3 Department of Chemistry, College of Science, King Saud University, Riyadh 11451, Saudi Arabia; krais@ksu.edu.sa

**Keywords:** Ti_3_AlC_2_, MAX phase, Ti_3_AlC_2_@SPCE, electrochemical hydrazine sensor

## Abstract

Hydrazine is considered a powerful reducing agent and catalyst, showing diverse applications in agricultural industries, toxic degradation research, and wastewater management. Additionally, hydrazine can trigger some specific reactions when combined with suitable oxidants. Due to its highly polar nature, hydrazine can easily dissolve in alcohol, water, and various other polar solvents. Therefore, it can be extensively utilized in different areas of application and industries such as rocketry and various chemical applications. Despite its beneficial properties, hydrazine is unstable, posing significant risk due to its highly toxic nature. It is extremely hazardous to both human health and the environment. It can cause various illnesses and symptoms such as dizziness, temporary blindness, damage to the central nervous system, and even death when inhaled in sufficient quantities. Therefore, it is highly important to monitor the level of hydrazine to prevent its toxic and hazardous effects on human beings and the environment. In the present study, we discuss the simple fabrication of a disposable cost-effective and eco-friendly hydrazine sensor. We used a screen-printed carbon electrode, i.e., SPCE, as a base for the construction of a hydrazine sensor. The Ti_3_AlC_2_ MAX has been used as a suitable and efficient electrode material for the fabrication of disposable hydrazine sensors. We modified the active surface of the SPCE using a drop-casting approach. The resulting Ti_3_AlC_2_ MAX modified SPCE (Ti_3_AlC_2_@SPCE) has been utilized as an efficient and low-cost hydrazine sensor. Cyclic voltammetry, i.e., CV, and linear sweep voltammetry, viz., LSV, was employed as a sensing technique in this study. The optimization of pH and electrode material loading was conducted. The Ti_3_AlC_2_@SPCE exhibited excellent sensing performance toward hydrazine oxidation. A reasonable detection limit (0.01 µM) was achieved for hydrazine sensing. The fabricated sensor also demonstrated a reasonable linear range of 1–50 µM. This work provides the design and fabrication of simple disposable Ti_3_AlC_2_@SPCE as a suitable electrode for the determination of hydrazine using LSV technology.

## 1. Introduction

Hydrazine is known for its highly reducing and powerful antioxidant properties, which makes it an outstanding reactive molecule. Hydrazine plays a crucial role in various industrial products and processes, such as pesticides, emulsifiers, catalysis, polymerization, explosives, and agriculture, due to its versatile nature [1,2]. It plays a key role in various applications, such as corrosion inhibition, plutonium extraction, rocket fuels, insecticides, pharmaceuticals, blowing agents, heat stabilization, photographic development, dyes, and plant growth regulation [3,4,5,6,7]. Although hydrazine has several benefits and applications in various fields, its highly toxic nature makes it hazardous [6,7]. Even a small amount of hydrazine exposure can cause various health problems in humans, such as burning eyes, vomiting, respiratory distress, blood-related disorders, unconsciousness, and skin damage [1,3]. However, prolonged exposure or inhalation of hydrazine may cause severe damage to vital organs like the kidneys and liver and may even increase the risk of cancer [2,6,7]. The related hazardous drawbacks associated with hydrazine have motivated researchers to either improve safety protocols or develop efficient hydrazine detection techniques [8,9,10,11,12]. Therefore, significant efforts have been carried out to develop highly sensitive methods for the accurate detection of hydrazine such as titration, chromatography, amperometry, potentiometry, spectrophotometry, and flow injection analysis [1,10,13,14,15,16,17,18,19]. These various methods and techniques emphasize the ongoing efforts to develop consistent and precise methods for the early and accurate detection of hydrazine. However, some inherent drawbacks associated with these methods, such as their time-consuming nature, high cost, need for sophisticated instruments, and limitations in detecting low levels of hydrazine, still present significant challenges [1,5,10,13]. Among various techniques, electrochemical methods are considered a promising approach to detecting hydrazine due to their high sensitivity, rapid response time, cost-effectiveness, ease of operation, and suitability for real-time detection [20,21,22]. While the electrochemical sensing techniques offer various advantages, they still face some drawbacks, such as insufficient conductivity and limited surface area, which hinder the development of an optimal sensor [7,23]. Additionally, optimizing the efficiency of electron transfer is vital for confirming rapid response times [5,20,22,23,24]. To improve the sensing capability of electrochemical techniques, it is necessary to modify surface properties and conductivity [24,25,26]. Therefore, various nanomaterials, such as copper oxide (CuO), zinc oxide (ZnO), iron oxide (Fe_2_O_3_), tin oxide (TiO_2_), tungsten oxide (WO_3_), and manganese dioxide (MnO_2_), and layered materials, have been investigated for the fabrication of electrochemical-based hydrazine chemical sensors and other applications [5,16,27,28,29,30,31,32,33,34,35,36]. Recently, MXene, an innovative two-dimensional material (2D), has gained significant attention across various fields, including electrochemical sensors, biofuel cell design, environmental remediation, bioimaging, biosensors, photothermal therapy, drug delivery, theranostics, antibacterials, and cytocompatibility [18,34,35]. In 2011, Professor Gogotsi and colleagues discovered MXenes, which exhibit remarkable electrical conductivity and a hydrophilic surface [37]. Due to their outstanding properties, such as a large specific surface area, abundant surface functional groups, excellent stretchability, and biocompatibility, MXenes emerge as a leading choice for the development of electrochemical sensors [38,39,40]. MXenes are characterized by the general formula M_n+1_AX_n_T_X_ where n ranges from 1 to 3, M denotes the transition metal, X represents either carbon or nitrogen, and Tx signifies absorbed species like -OH, -O, and -F on the surface [41,42,43,44]. First, MXene was synthesized by selectively etching element A with hydrofluoric acid (HF) solution, leading to the formation of surface functional groups denoted as T_X_ [37]. The composition and properties of these functional groups vary depending on the chosen etching route [45,46]. Titanium carbide (Ti_3_C_2_T_X_) is notably the first reported MXene and has been extensively studied within the MXene family. This makes it a prime candidate for various electrochemical sensor applications, including glucose detection, hydrazine sensing, environmental pesticide monitoring, and pharmaceutical compound sensing [34,37,45]. Significantly, Ti_3_C_2X_ demonstrates remarkably high metallic conductivities ranging from 6000 to 8000 S/cm, surpassing many other 2D layered materials [45,47,48]. It is a good substrate for developing electrochemical sensing technology for hydrazine because of its remarkable intrinsic electrical conductivity and reactive hydrophilic surface [38,49]. Ti_3_C_2_T_X_ is expected to perform better in electrochemical sensing applications than other 2D layered nanostructures [40,43].

In this study, we have developed an electrochemical sensor that exhibits a low limit of detection (LOD) for hydrazine with high sensitivity. Ti_3_AlC_2_ MAX phase and a modified screen-printed electrode assembly approach were utilized to fabricate an effective and low-cost hydrazine sensor. Notably, controlled exfoliation of Ti_3_AlC_2_ MAX was performed without using any external surface treatment. This approach improves electrochemical and catalytic properties, resulting in increased sensitivity and a lower detection limit for hydrazine. Considering the hazardous nature of toxic hydrazine, a cautious approach was employed for its detection. A working electrode for hydrazine detection was fabricated by depositing a Ti_3_AlC_2_ MAX layer onto a screen-printed carbon electrode (SPCE) with Nafion as a binder. The electrochemical sensor reported in this work demonstrates the ability to detect hydrazine with a low detection limit (DL) while also exhibiting good sensitivity, selectivity, reproducibility, stability, and repeatability. So far, no report is available on the fabrication of Ti_3_AlC_2_ MAX layer-based hydrazine sensor. This is the first report that used the Ti_3_AlC_2_ MAX layer-coated SPCE as a hydrazine sensor. 

## 2. Materials and Methods

### 2.1. Chemicals and Reagents

Dopamine, uric acid, glucose, cholesterol, hydrogen peroxide, urea, sodium chloride, magnesium chloride, cadmium chloride, mercury chloride, catechol, ammonia, 4-chlorophenol, 4-nitrophenol, methanol, and acetone were obtained from Merck. Phosphate buffer saline (PBS) was purchased from Sigma Aldrich. 

### 2.2. Apparatus

The powder X-ray diffraction (P-XRD) pattern of the Ti_3_AlC_2_ MAX was obtained on Rigaku company (Japan), RINT 2500 V X-ray diffractometer with Cu/Kα irradiation (λ = 1.5406 Å). The surface topological results, i.e., scanning electron microscopic (SEM) images of the Ti_3_AlC_2_ MAX, were recorded on a field emission scanning electron microscope (FESEM; Supra 55 Zeiss) equipped with energy-dispersive X-ray (EDAX) spectroscopy (Oxford Instrument’ X-max; Aztec). The EDAX spectrum of the Ti_3_AlC_2_ MAX was examined using Oxford Instrument’ X-max, an Aztec instrument. The electrochemical sensing studies were carried out on Metrohm Autolab PGSTAT 302N (Nova software has been used to record the data; version 1.10) instrument. The screen-printed carbon electrode, i.e., SPCE, was used as a working electrode, while a silver/silver chloride (Ag/AgCl) electrode was utilized as a reference electrode. The platinum wire-based counter electrode was employed as a counter electrode. The electrochemical measurements were performed in a three-electrode assembly system. 

### 2.3. Fabrication of Sensor

**Scheme 1:**
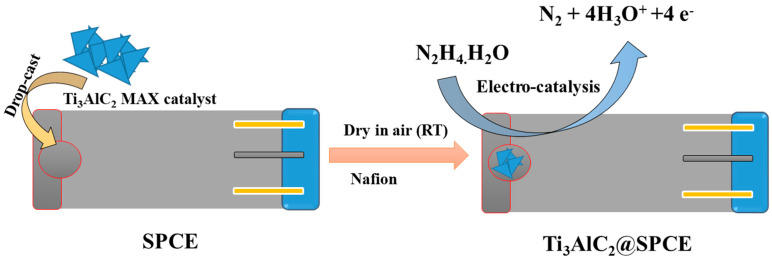
The schematic representation for the construction of Ti_3_AlC_2_@SPCE for hydrazine detection.

The Ti_3_AlC_2_ MAX (3 mg/mL) was sonicated in deionized water (DI) for a few hours to obtain the homogenous dispersion of the Ti_3_AlC_2_ MAX. Nafion (binder), 0.5 wt%, was added to the Ti_3_AlC_2_ MAX dispersion and sonicated for another 1 h. The bare active surface of the SPCE was modified using Ti_3_AlC_2_ MAX as electrode material. Different amounts of Ti_3_AlC_2_ MAX dispersion (4 µL, 6 µL, 8 µL, and 10 µL) were drop-cast onto the active surface of the SPCE and let dry for a few hours. 7.5 µL of the glucose oxidase was also coated on the fabricated electrode to improve the catalytic activity. The Ti_3_AlC_2_ MAX modified (after drying process) was used as a working electrode for electrochemical sensing application. In the paper, the Ti_3_AlC_2_ MAX modified SPCE electrode was denoted as Ti_3_AlC_2_@SPCE. The surface modification and operation of the Ti_3_AlC_2_@SPCE for hydrazine sensing are illustrated in Scheme 1 below. 

## 3. Results and Discussion

### 3.1. Materials Characterization

**Figure 1:**
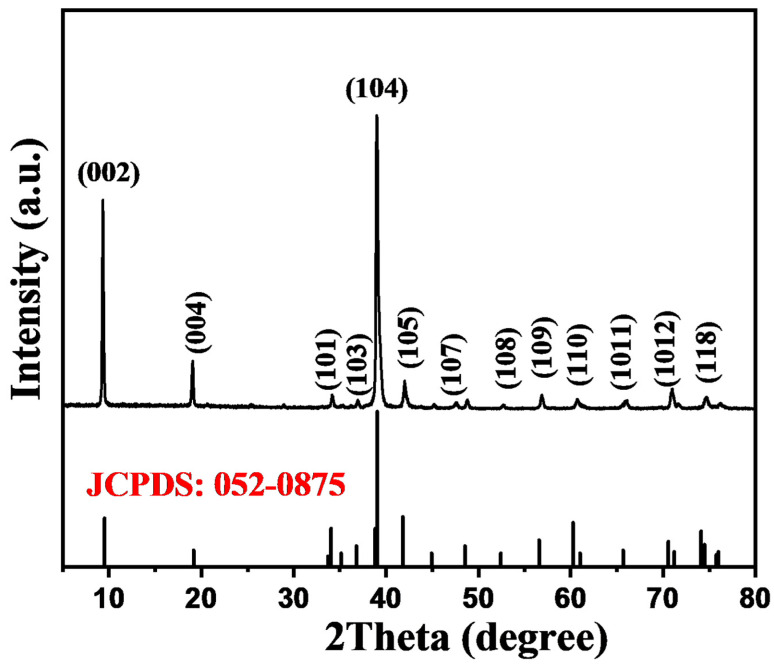
P-XRD spectrum of the Ti_3_AlC_2_ MAX.

The phase purity and crystalline nature can be determined using the P-XRD technique [50,51]. In this study, the P-XRD pattern of the Ti_3_AlC_2_ MAX was obtained in the applied 2theta range of 5–80°. Figure 1 illustrates the P-XRD spectrum of Ti_3_AlC_2_ MAX. The P-XRD pattern of the Ti_3_AlC_2_ MAX shows three major diffraction peaks observed at 2theta values of 9.17°, 18.98°, and 38.97°, which can be assigned to the (002), (004), and (104) diffraction planes, respectively. 

**Figure 2:**
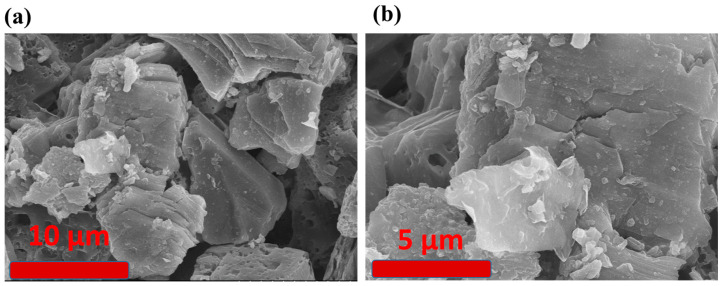
(**a**,**b**) FE-SEM images of the Ti_3_AlC_2_ MAX.

However, other diffraction peaks at 2theta value of 34.15°, 36.83°, 41.96°, 47.61°, 52.67°, 56.88°, 60.71°, 65.97°, 70.93°, and 74.54° correspond to (101), (103), (105), (107), (108), (109), (110), (1011), (1012), and (118) diffraction planes of Ti_3_AlC_2_ MAX, respectively. This P-XRD pattern and diffraction planes are in good agreement with the reported JCPDS-number 052-0875. The presence of strong diffraction peaks with high intensity indicates the presence of a good crystalline nature while the absence of any additional diffraction peak related to the impurities suggests a high degree of phase purity. Surface morphological properties also play a key role in determining the electrochemical sensing performance. Therefore, it is important to examine the surface morphological characteristics of the Ti_3_AlC_2_ MAX. The obtained SEM images are displayed in Figure 2a,b. The SEM results show that Ti_3_AlC_2_ MAX has a flake-like structure that forms the sheets. 

The Ti_3_AlC_2_ MAX is a two-dimensional (2D) material, and SEM images show that Ti_3_AlC_2_ MAX possesses a sheet-like surface structure, which may be beneficial for better electron transport during the redox reactions at the surface of the fabricated electrochemical sensors [52]. It is known that flake-like surface-structured materials possess a high surface area, which may provide a better path for charge transport along the plane of the flakes. This can lead to faster electron transfer compared with bulk materials where charge transport may be hindered by longer pathways or structural defects. It is also expected that flake-like morphology can improve the contact between the material and the analyte in three electrochemical systems. This may benefit the exchange of ions or molecules involved in the redox reactions, leading to improved electron transport. Thus, it is believed that Ti_3_AlC_2_ MAX would be beneficial for electrochemical sensing applications. 

**Figure 3:**
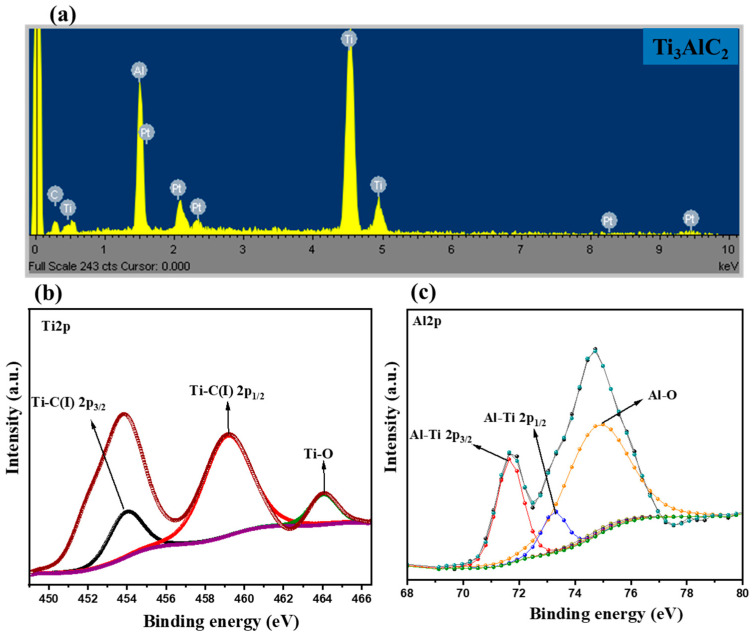
(**a**) EDX spectrum of the Ti_3_AlC_2_ MAX. (**b**) Ti2p and (**c**) Al2p XPS scan of Ti_3_AlC_2_ MAX.

Furthermore, the elemental composition and phase purity of the Ti_3_AlC_2_ MAX were studied using the EDAX technique. The EDAX spectrum of the Ti_3_AlC_2_ MAX was obtained using an EDAX spectroscope. Figure 3a displays the EDAX spectrum of the Ti_3_AlC_2_ MAX. The EDAX spectrum indicates the signals for C, Ti, and Al elements. It can be clearly understood that Ti_3_AlC_2_ MAX has good phase purity. 

Furthermore, elemental oxidation states of the Ti2p and Al2p of the Ti_3_AlC_2_ MAX have been studied using X-ray photoelectron spectroscopy (XPS). The obtained XPS results for the Ti2p and Al2p XPS scans are provided in Figure 3b,c. Figure 3b shows the XPS scan of Ti2p, which exhibits the presence of three major XPS signals at binding energy values of 454.01, 459.23, and 464.2 eV, which can be assigned to the presence of Ti-C(I) 2p_3/2_, Ti-C(I) 2p_1/2_ and Ti-O bonds, respectively. Similarly, Figure 3c shows a high-resolution scan of Al2p XPS which reveals the presence of three de-convoluted XPS peaks at binding energy values of 71.67, 73.30, and 74.87 eV, which can be ascribed to the presence of Al-Ti 2p_3/2_, Al-Ti 2p_1/2_ and Al-O bonds, respectively [53]. 

### 3.2. Electrochemical Performance

**Figure 4:**
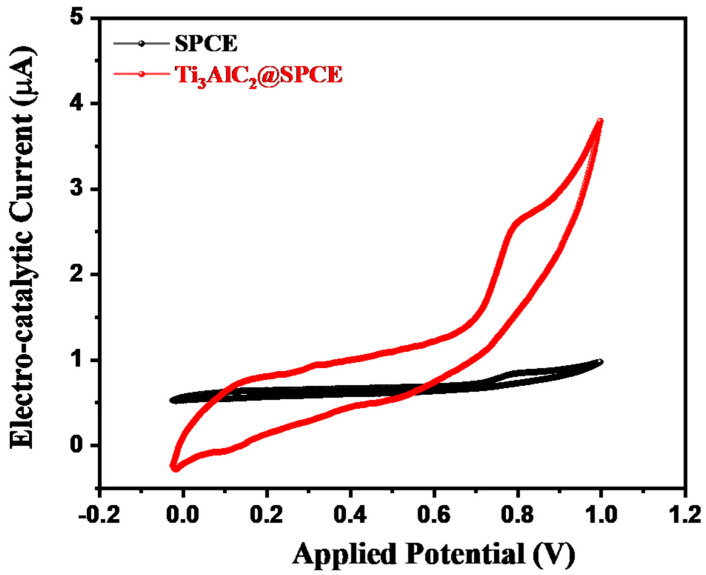
CVs of the bare SPCE (black line curve) and Ti_3_AlC_2_@SPCE (red line curve) in the presence of 55 µM Hz under the 0.1 M PBS conditions (pH = 8.0) at the applied scan potential rate of 50 mVs^−1^.

After physiochemical characterization of the Ti_3_AlC_2_ MAX, a based sensor was fabricated using Ti_3_AlC_2_ MAX as a catalyst to facilitate electron transport during the oxidation of hydrazine. The carbon-based bare surface of the SPCE was modified with Ti_3_AlC_2_ MAX as an electrocatalyst via drop-casting the dispersion (8 µL) of the Ti_3_AlC_2_ MAX. Cyclic voltammetry (CV) is one of the basic electrochemical techniques to study the electrochemical behavior or properties of the modified electrodes in a particular analyte. Thus, it is important to study the electrochemical behavior of the bare SPCE and Ti_3_AlC_2_@SPCE for the oxidation of hydrazine (Hz). In this context, the CV approach was utilized for the electrochemical oxidation of Hz. In the first step, the CV of the bare SPCE was recorded for the electrochemical oxidation of 55 µM Hz under the 0.1 M PBS conditions (pH = 8.0) at the applied scan potential rate of 50 mVs^−1^, as shown in Figure 4 (black color pattern). 

The obtained results show a very low response in terms of the current value. The bare SPCE exhibited poor catalytic behavior for the oxidation of Hz using the CV method. Further, we have obtained the CV pattern of the Ti_3_AlC_2_@SPCE in the presence of 55 µM Hz under the 0.1 M PBS conditions (pH = 8.0) at the applied scan potential rate of 50 mVs^−1^. The obtained CV graph is shown in Figure 4. According to the obtained CV results, it can be seen from Figure 4 that Ti_3_AlC_2_@SPCE shows good catalytic properties in comparison to the bare SPCE. The Ti_3_AlC_2_@SPCE shows an enhanced current response—in comparison to the bare SPCE—to the oxidation of 55 µM Hz under the 0.1 M PBS conditions (pH = 8.0) at the applied scan potential rate of 50 mVs^−1^. Therefore, it is clear that a significant improvement in the oxidation of Hz is observed, which may be attributed to the presence of conductive properties of the Ti_3_AlC_2_ on the surface of the modified SPCE. The aforementioned CV results have been studied for an 8 µL catalyst (Ti_3_AlC_2_ MAX). The amount of catalyst can significantly affect the catalytic properties of the developed sensors or electrodes toward the electro-oxidation of Hz. Thus, the loading amount of the Ti_3_AlC_2_ MAX was optimized toward the sensing of 55 µM Hz under the 0.1 M PBS conditions (pH = 8.0) at the applied scan potential rate of 50 mVs^−1^. Figure S1 shows the current responses of the Ti_3_AlC_2_@SPCE (loaded with different amounts of 4 µL, 6 µL, 8 µL, and 10 µL) toward the oxidation of 55 µM Hz. It can be seen from Figure S1 that first, the current value increases up to 8 µL, and then, a reduction is observed at 10 8 µL. Among the different loading amounts, 8 µL showed the higher current value for modified Ti_3_AlC_2_@SPCE toward the oxidation of 55 µM Hz. Thus, 8 µL modified Ti_3_AlC_2_@SPCE was used for all the further electrochemical studies. The pH of the analyte solution may also play a significant role and should be optimized. Hence, we have optimized the pH of the analyte solution. In this context, current responses of the Ti_3_AlC_2_@SPCE were also obtained for 55 µM Hz under the 0.1 M PBS conditions (pH = 2.0, 4.0, 6.0, 8.0, and 10.0) at the applied scan potential rate of 50 mVs^−1^. The current responses are summarized in Figure S2. The observations indicate that Ti_3_AlC_2_@SPCE has a good electrocatalytic response for 55 µM Hz in the presence of 0.1 M PBS at pH 8.0. Therefore, it is reasonable to use 0.1 M PBS at pH 8.0 for further investigations. Hence, 0.1 M PBS of pH 8.0 was used as an optimized condition for other electrochemical examinations. 

**Figure 5:**
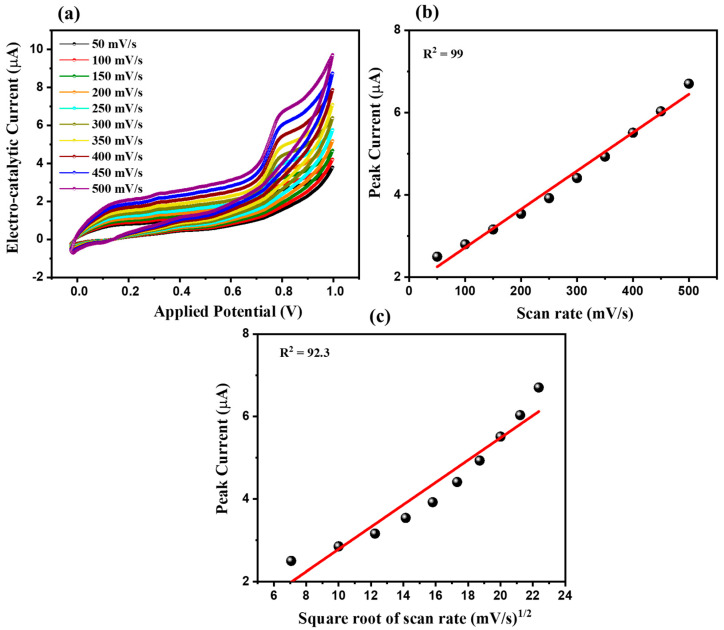
(**a**) CVs of the Ti_3_AlC_2_@SPCE in the presence of 55 µM Hz under the 0.1 M PBS conditions (pH = 8.0) at different applied scan potential rates (50 mVs^−1^, 100 mVs^−1^, 150 mVs^−1^, 200 mVs^−1^, 250 mVs^−1^, 300 mVs^−1^, 350 mVs^−1^, 400 mVs^−1^, 450 mVs^−1^, and 500 mVs^−1^). (**b**) Calibration curve of the oxidation peak current values against the applied scan rate. (**c**) The calibration curve between the oxidation peak current values with respect to the square root of the applied scan rate.

The applied scan rate of the electrochemical setup may affect the current responses of the working electrode toward the diffusion or adsorption of the analyte at a particular potential. The CV response of the Ti_3_AlC_2_@SPCE was recorded in the presence of 55 µM Hz under the 0.1 M PBS conditions (pH = 8.0) at different applied scan potential rates (50 mVs^−1^, 100 mVs^−1^, 150 mVs^−1^, 200 mVs^−1^, 250 mVs^−1^, 300 mVs^−1^, 350 mVs^−1^, 400 mVs^−1^, 450 mVs^−1^, and 500 mVs^−1^). The obtained CV results at different applied scan rates are summarized in Figure 5a, which shows that the current response of the Ti_3_AlC_2_@SPCE increases as the applied scan rates. 

In order to see the effect of different scan rates, we have plotted the calibration curve between the current response and the applied scan rate as shown in Figure 5b. It can be seen from Figure 5b that the current response of the Ti_3_AlC_2_@SPCE for 55 µM Hz linearly increases with scan rates. Furthermore, the regression constant (R^2^) value of 0.99 is obtained from the calibration curve after fitting the data, which suggests that the electrochemical oxidation of Hz is an adsorption-controlled process rather than a diffusion-controlled process. The calibration plot of the current response of the Ti_3_AlC_2_@SPCE with respect to the square root of the applied scan rates is presented in Figure 5c. The R^2^ value of 92.3 is obtained, which also supports that the electrochemical oxidation of Hz is an adsorption-controlled process. Linear sweep voltammetry, i.e., LSV, method can be a more sensitive approach compared with the CV. Using the simple and sensitive linear sweep voltammetry (LSV) approach as a sensing platform for detecting Hz is worthwhile.

**Figure 6:**
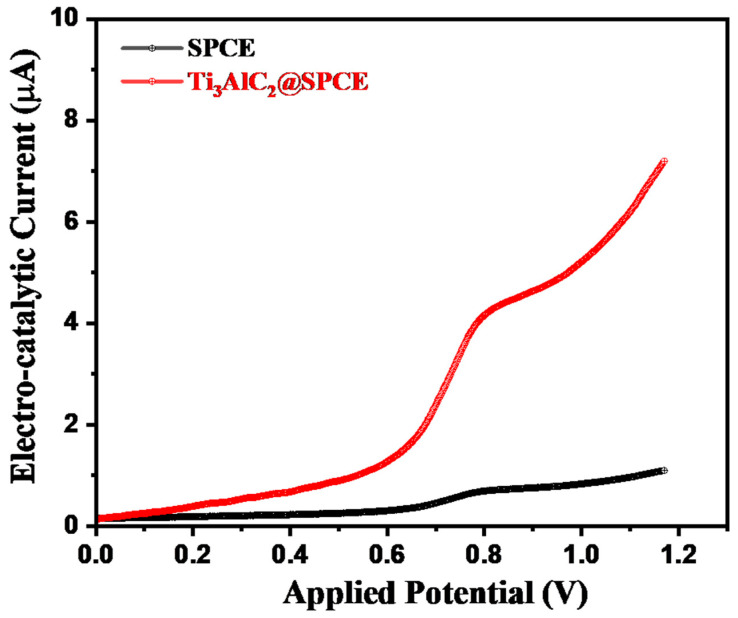
LSVs of the bare SPCE (black line curve) and Ti_3_AlC_2_@SPCE (red line curve) in the presence of 55 µM Hz under the 0.1 M PBS conditions (pH = 8.0) at the applied scan potential of 50 mVs^−1^.

Therefore, we have used the LSV approach to further the sensing platform for the electrochemical oxidation of Hz. The LSV patterns of the bare SPCE and Ti_3_AlC_2_@SPCE were collected in the presence of 55 µM Hz under the 0.1 M PBS conditions (pH = 8.0) at the applied scan potential of 50 mVs^−1^. The obtained LSV results are given in Figure 6. It can be clearly observed that bare SPCE has a poor current response signal for the electrochemical oxidation of Hz. In the case of the electrochemical oxidation of Hz using Ti_3_AlC_2_@SPCE, an improved LSV signal is observed, as shown in Figure 6. This indicates that Ti_3_AlC_2_@SPCE exhibits higher catalytic features in the electrochemical oxidation of Hz compared with SPCE under similar conditions. 

**Figure 7:**
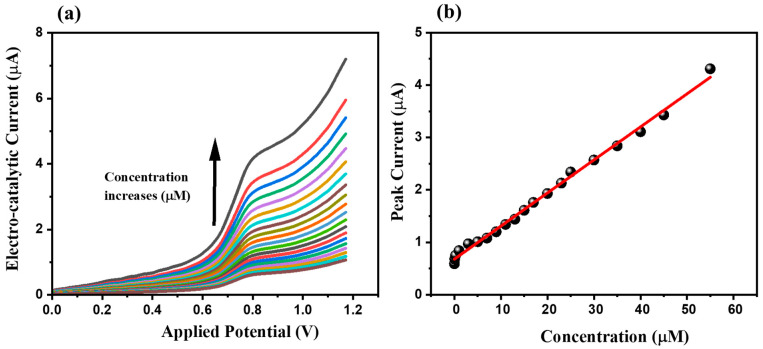
(**a**) LSV graphs of the Ti_3_AlC_2_@SPCE in the presence of different concentrations (0.01 µM, 0.08 µM, 0.25 µM, 1 µM, 3 µM, 5 µM, 7 µM, 9 µM, 11 µM, 13 µM, 15 µM, 17 µM, 20 µM, 23 µM, 25 µM, 30 µM, 35 µM, 40 µM, 45 µM, and 55 µM) of Hz under the 0.1 M PBS conditions (pH = 8.0) at the applied scan potential of 50 mVs^−1^. (**b**) The calibration plot shows the linear relation between the oxidation peak current response and the concentration of Hz.

In further experiments, various concentrations of Hz have been utilized to investigate the electrochemical detection of Hz at low-level concentrations. In this regard, LSV patterns of the Ti_3_AlC_2_@SPCE were recorded in the presence of different concentrations (0.01 µM, 0.08 µM, 0.25 µM, 1 µM, 3 µM, 5 µM, 7 µM, 9 µM, 11 µM, 13 µM, 15 µM, 17 µM, 20 µM, 23 µM, 25 µM, 30 µM, 35 µM, 40 µM, 45 µM, and 55 µM) of Hz under the 0.1 M PBS conditions (pH = 8.0) at the applied scan potential of 50 mVs^−1^. The LSV patterns of the Ti_3_AlC_2_@SPCE in various concentrations of Hz are summarized in Figure 7a. It can be seen that the current response value of the Ti_3_AlC_2_@SPCE toward the electrochemical oxidation of Hz increases with concentration (0.01 µM, 0.08 µM, 0.25 µM, 1 µM, 3 µM, 5 µM, 7 µM, 9 µM, 11 µM, 13 µM, 15 µM, 17 µM, 20 µM, 23 µM, 25 µM, 30 µM, 35 µM, 40 µM, 45 µM, and 55 µM) of the Hz at the fixed applied potential scan rate. The linear calibration plot between the current response values of the Ti_3_AlC_2_@SPCE and the concentration of the Hz is shown in Figure 7b. The linear calibration curve shows the R^2^ value of 0.99, which suggests that the current response to the electrochemical oxidation of Hz linearly increases. 

**Figure 8:**
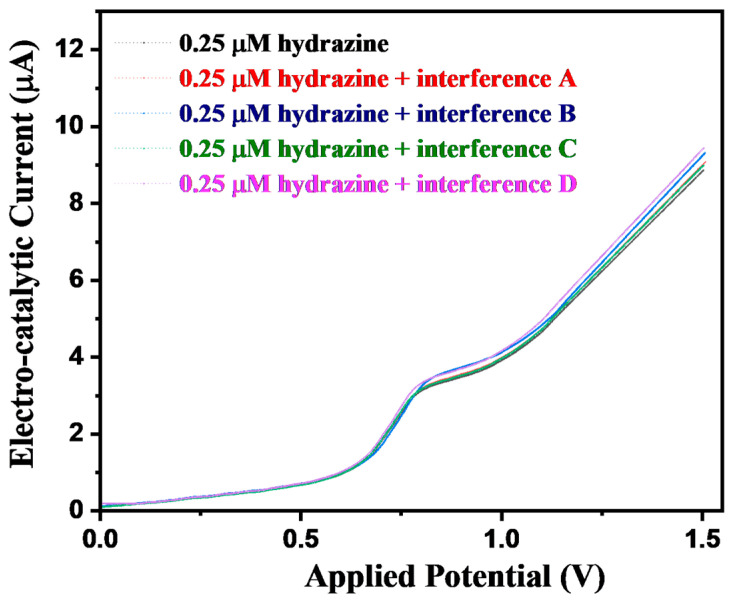
LSVs of the Ti_3_AlC_2_@SPCE in the presence of 0.25 µM Hz, 0.25 µM Hz + 1 µM interference A (DA, UA, CA, glucose, urea), 0.25 µM Hz + 1 µM interference B (H_2_O_2_, ammonia, 4-chlorophenol, 4-nitrophenol, cholesterol), 0.25 µM Hz + 1 µM interference C (Na^+^, Cl^−^, Mg^2+^, Cd^2+^, Hg^2+^), and 0.25 µM Hz + 1 µM interference D (catechol, methanol, acetone) under the 0.1 M PBS conditions (pH = 8.0) at the applied scan potential of 50 mVs^−1^.

In the present world, various similar electro-active materials and compounds exist, which may affect the accurate detection of Hz. Therefore, a sensor should possess selectivity for a specific analyte. Since a three-electrode assembly operates in a liquid electrolyte system, the presence of other electro-active materials may impact the precise determination of Hz using Ti_3_AlC_2_@SPCE. In this context, we investigated the selectivity studies using the LSV method. The standard solutions of the electro-active materials were prepared in four batches (A= dopamine (DA), uric acid (UA), citric acid (CA), glucose, urea); B = (H_2_O_2_, ammonia, 4-chlorphenol, 4-nitrophenol, cholesterol), C= (Na^+^, Cl^−^, Mg^2+^, Cd^2+^, Hg^2+^) and D = (catechol, methanol, acetone)). The LSV responses of the Ti_3_AlC_2_@SPCE were obtained in the presence of 0.25 µM Hz, 0.25 µM Hz + 1 µM interference A (DA, UA, CA, glucose, urea), 0.25 µM Hz + 1 µM interference B (H_2_O_2_, ammonia, 4-chlorophenol, 4-nitrophenol, cholesterol), 0.25 µM Hz + 1 µM interference C (Na^+^, Cl^−^, Mg^2+^, Cd^2+^, Hg^2+^), and 0.25 µM Hz + 1 µM interference D (catechol, methanol, acetone) under the 0.1 M PBS conditions (pH = 8.0) at the applied scan potential of 50 mVs^−1^. Figure 8 shows that the presence of various interfering materials did not significantly affect the performance of the Ti_3_AlC_2_@SPCE and suggests its selective properties for the detection of Hz. 

**Figure 9:**
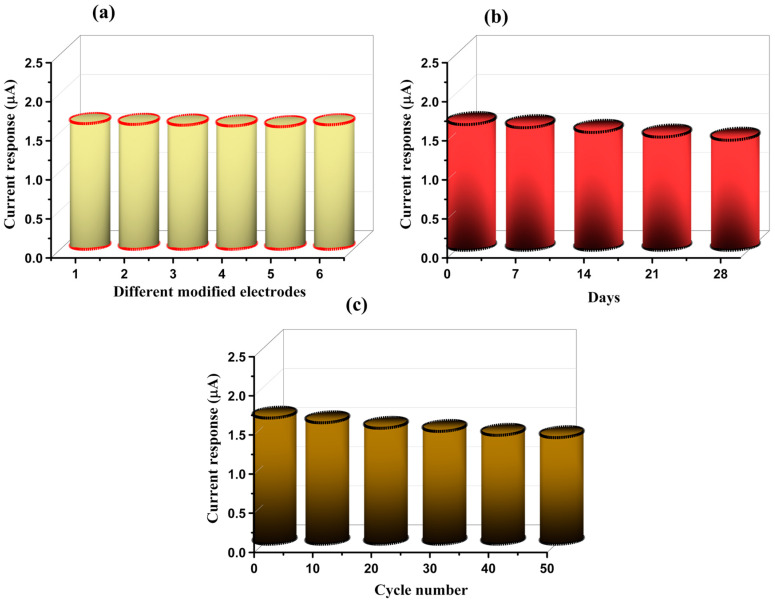
(**a**) Reproducibility study, (**b**) storage stability study, and (**c**) repeatability study for Ti_3_AlC_2_@SPCE in the presence of 0.25 µM Hz under the 0.1 M PBS conditions (pH = 8.0) at the applied scan potential rate of 50 mVs^−1^.

Reproducibility, repeatability, and storage stability are the most desirable features in electrochemical sensors. To evaluate these aspects, six different SPCEs were fabricated under similar environments and optimized conditions. Their performance was investigated for 0.25 µM Hz under the 0.1 M PBS conditions (pH = 8.0) at the applied scan potential rate of 50 mVs^−1^. It can be seen from Figure 9a that there is hardly a noticeable change observed, which suggests its good reproducibility. The Ti_3_AlC_2_@SPCE also showed good storage stability of 28 days as shown in Figure 9b. The repeatability study also investigated 0.25 µM Hz under the 0.1 M PBS conditions (pH = 8.0) at the applied scan potential rate of 50 mVs^−1^. Figure 9c demonstrates the presence of good repeatability for 50 cycles. 

The probable electrochemical sensing mechanism for the oxidation of Hz is illustrated below.

(a) N_2_H_4_ + H_2_O → N_2_H_3_ + H_3_O^+^ + e^−^ (slow rate-determining step) (1)

(b) N_2_H_3_ + 3H_2_O → N_2_ + 3H_3_O^+^ + 3e^−^ (fast-rate step) (2)

(c) N_2_H_4_ + 4H_2_O → N_2_ + 4H_3_O^+^ + 4e^−^ (overall reaction) (3)

The first step is a slow step, which may generate the N_2_H_3_ and H_3_O^+^ ions in the oxidation of Hz. The second step, which is the last step, may generate nitrogen. The overall reaction for the oxidation of Hz can be seen in Equation (3). 

The detection limit, i.e., DL, of the Ti_3_AlC_2_@SPCE is determined by utilizing the formula as listed in Equation (4),

DL = 3.3 × σ_s_/slope (4)

In addition, the sensitivity of the Ti_3_AlC_2_@SPCE is calculated by employing the formula as listed in Equation (5),

Sensitivity = Slope value/Area of the e Ti_3_AlC_2_@SPCE (5)

The DL of the Ti_3_AlC_2_@SPCE for the oxidation of Hz has been compiled in Table 1 with previous reports on the sensing of Hz. Previously, various chemically modified electrodes have been reported for detecting Hz. In this context, the ZrO_2_ NPs/Au electrode was reported as a Hz sensor by Bansal et al. [42] whereas a WO_3-_based Hz sensor was reported by Shukla et al. [27]. Cr_2_CT_x_ MXene was also used as the electrocatalyst for the fabrication of the Hz sensor [44]. Previously, various nanostructured materials were explored as the electrocatalysts for the fabrication of Hz sensors as shown in Table 1. The reported articles in Table 1 demonstrated decent performance, but most of the Hz sensors suffer from poor stability over long-term cycles and poor conductivity of the metal oxides such as WO_3_. Some of the Hz sensors involve the use of high-cost Au electrodes, which is another challenge for the development of cost-effective Hz sensors. Thus, it is necessary to develop a low-cost and highly sensitive Hz sensor with improved stability and performance. In this study, we reported the construction of a Hz sensor using conductive Ti_3_AlC_2_ as a catalyst and disposable SPCE as a working electrode. The obtained results in our present study are reasonably good compared with the recent reports listed in Table 1 [22,27,30,31,36,42,44,52,54,55,56,57,58,59,60]. 

## 4. Conclusions

In conclusion, it can be summarized that Ti_3_AlC_2_@SPCE was modified using a simple drop-cast method. The phase purity and crystalline nature of the Ti_3_AlC_2_ MAX were determined using P-XRD analysis. The P-XRD study indicates the presence of good crystallinity and phase purity of the Ti_3_AlC_2_ MAX. Additionally, surface properties such as morphological characteristics of the Ti_3_AlC_2_ MAX were investigated using the SEM method. The SEM results suggested the presence of a sheet-like structure of the Ti_3_AlC_2_ MAX. Furthermore, elemental composition was confirmed by employing the EDAX technique. Finally, Ti_3_AlC_2_@SPCE was developed using Ti_3_AlC_2_ MAX as an electrocatalyst for the fabrication of the Hz sensor. The Ti_3_AlC_2_@SPCE demonstrated good detection limit, wide dynamic linear range, and sensitivity. The Ti_3_AlC_2_@SPCE also exhibited decent selectivity for the determination of Hz in the presence of various interfering molecules or salts. The Ti_3_AlC_2_@SPCE also exhibited good storage stability over 28 days. Furthermore, Ti_3_AlC_2_@SPCE demonstrated reasonable repeatability stability over 50 cycles. This work reports the simple fabrication of a cost-effective eco-friendly, and disposable Ti_3_AlC_2_@SPCE for the determination of Hz using the LSV method. 

## Figures and Tables

**Table 1:** Comparison of the LOD, linear range, and sensitivity of the Ti_3_AlC_2_@SPCE with previous studies.

Electrode	DL (µM)	Linear Range (µM)	Sensitivity (µA/µM.cm^2^)	References
Ti_3_AlC_2_@SPCE	0.01	1–50	6.76	This study
ZrO_2_ NPs/Au electrode	1.05	-	8.99	[42]
WO_3_	144	-	-	[27]
Cr_2_CT_x_ MXene	0.66	1.99–200	1.81	[44]
ZnO	2.1	-	-	[52]
Erbium-doped Nb_2_CT_x_	67	-	-	[44]
PVP-AgNCs/GCE	1.1	-	-	[22]
CuO nanosheets (CuO NSs)/Cellulose acetate butyrate (CAB)	0.15	-	-	[30]
SnO_2_/ZnO	0.36	2.5–25	1.16	[31]
Au NPs/MnO_2_ Composites	1.7	-	-	[36]
Au@ carbon nano-onions/GCE	0.012	0.05–1000	-	[54]
MoS_2_/rGO/Au	0.5	2–30	-	[55]
Ionic liquid/CoS_2_-CNT/CP	0.015	0.03–500	0.073	[56]
MoS_2_-QDs@Fe_3_O_4_/rGO	0.12	0.8–2190	0.035	[57]
rGO-MoS_2_	0.132	-	89.89	[58]
PANI-MoS_2_	0.40	10–100	7.23	[59]
TiO_2_@PANI/GCE	0.15	-	-	[60]

## Data Availability

Data can be available on reasonable request.

## Supplementary Materials

The following supporting information can be downloaded at: https://www.mdpi.com/article/10.3390/mi15050633/s1.
