# Supplementary material for: Ti3AlC2 MAX Phase Modified Screen-Printed Electrode for the Fabrication of Hydrazine Sensor
Source: Micromachines (Basel). 2024 May 9;15(5):633. doi: 10.3390/mi15050633 (PMC11122756; doi:10.3390/mi15050633)
Supplement: Supplementary file 1 [file micromachines-15-00633-s001.zip › micromachines-2963331-supplementary.pdf]

Supporting Information

Ti_3AlC_2 MAX Phase Modified Screen-Printed Electrode for the Fabrication of Hydrazine Sensor

Khursheed Ahmad [1,*], Waseem Raza [2] and Rais Ahmad Khan [3]

[1] School of Materials Science and Engineering, Yeungnam University, Gyeongsan 38541, Republic of Korea
[2] Department of Materials Science and Engineering, WW4-LKO, University of Erlangen-Nuremberg, Martensstrasse 7, 91058 Erlangen, Germany
[3] Department of Chemistry, College of Science, King Saud University, Riyadh 11451, Saudi Arabia
[*] Correspondence: khursheed.energy@gmail.com

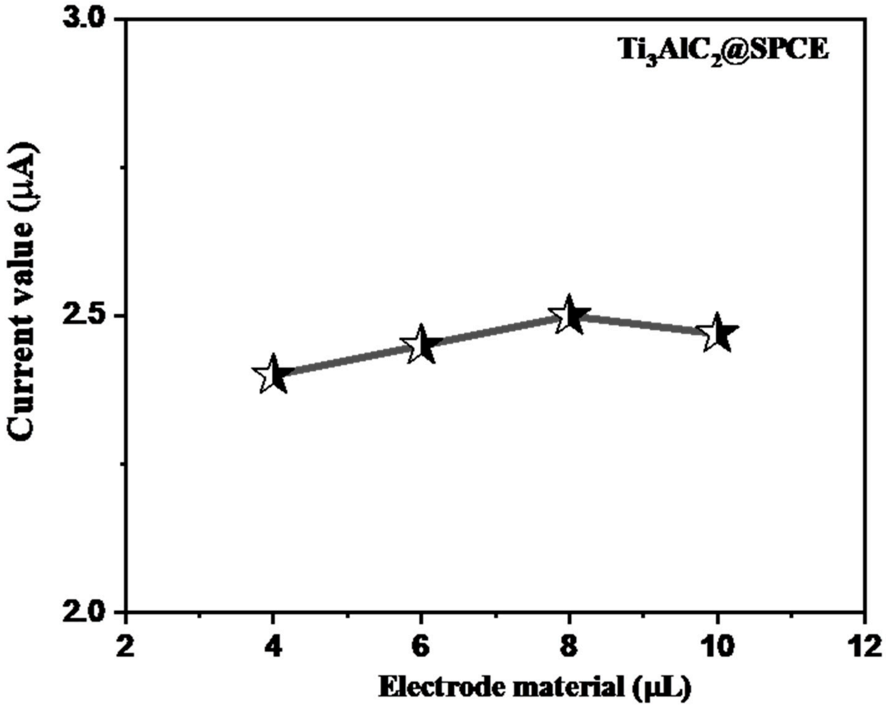

Figure S1. Current responses of the different mass loading of the Ti_3AlC_2@SPCE in presence of 55 μM Hz under the 0.1 M PBS conditions (pH = 8.0) at the applied scan potential of 50 mVs^{-1}.

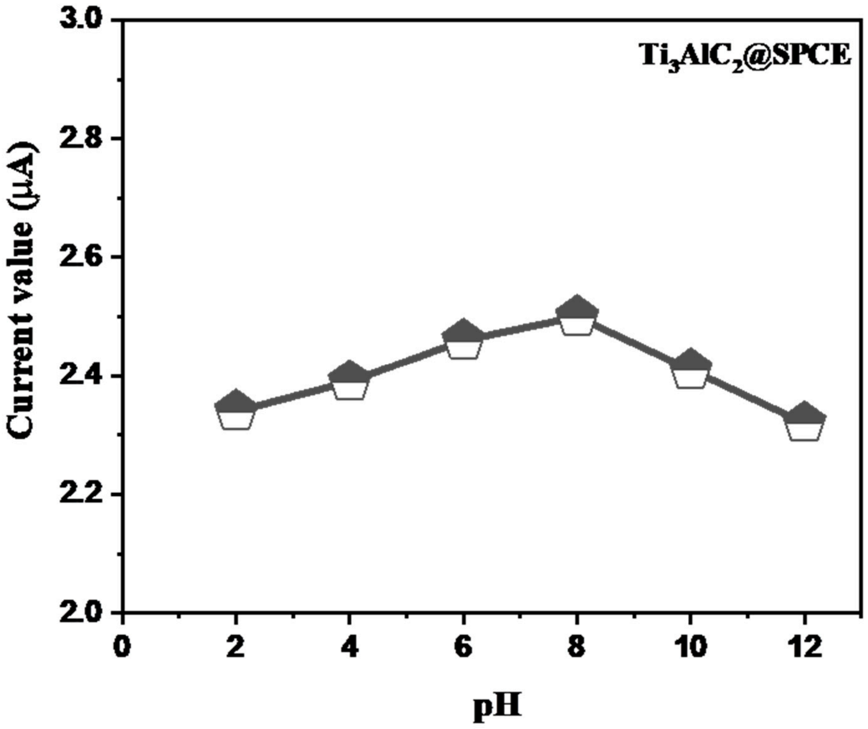

Figure S2. Current responses of the Ti_3AlC_2@SPCE in presence of 55 μM Hz under the 0.1 M PBS conditions (pH = 2, 4, 6, 8, 10, and 12) at the applied scan potential of 50 mVs^{-1}.
